# Expression, Purification, and Characterization of (*R*)-Sulfolactate Dehydrogenase (ComC) from the Rumen Methanogen *Methanobrevibacter millerae* SM9

**DOI:** 10.1155/2017/5793620

**Published:** 2017-11-06

**Authors:** Yanli Zhang, Linley R. Schofield, Carrie Sang, Debjit Dey, Ron S. Ronimus

**Affiliations:** AgResearch Limited, Grasslands Research Centre, Tennent Drive, Private Bag 11008, Palmerston North 4442, New Zealand

## Abstract

(*R*)-Sulfolactate dehydrogenase (EC 1.1.1.337), termed ComC, is a member of an NADH/NADPH-dependent oxidoreductase family of enzymes that catalyze the interconversion of 2-hydroxyacids into their corresponding 2-oxoacids. The ComC reaction is reversible and in the biosynthetic direction causes the conversion of (*R*)-sulfolactate to sulfopyruvate in the production of coenzyme M (2-mercaptoethanesulfonic acid). Coenzyme M is an essential cofactor required for the production of methane by the methyl-coenzyme M reductase complex. ComC catalyzes the third step in the first established biosynthetic pathway of coenzyme M and is also involved in methanopterin biosynthesis. In this study, ComC from *Methanobrevibacter millerae* SM9 was cloned and expressed in *Escherichia coli* and biochemically characterized. Sulfopyruvate was the preferred substrate using the reduction reaction, with 31% activity seen for oxaloacetate and 0.2% seen for *α*-ketoglutarate. Optimal activity was observed at pH 6.5. The apparent *K*_M_ for coenzyme (NADH) was 55.1 *μ*M, and for sulfopyruvate, it was 196 *μ*M (for sulfopyruvate the *V*_max_ was 93.9 *μ*mol min^−1^ mg^−1^ and *k*_cat_ was 62.8 s^−1^). The critical role of ComC in two separate cofactor pathways makes this enzyme a potential means of developing methanogen-specific inhibitors for controlling ruminant methane emissions which are increasingly being recognized as contributing to climate change.

## 1. Introduction

Coenzyme M is an essential cofactor for the final reaction in the methanogenesis pathway and in the production of methane catalyzed by methyl-coenzyme M reductase (MCR) [1]. It is the smallest known cofactor, acts during catalysis as a nucleophile [1, 2], and can reach millimolar concentrations within the cell [3, 4]. In *Methanobrevibacter ruminantium*, the uptake of CoM is an energy-dependent reaction [3, 5].

Recently, it has been found that the biosynthesis of coenzyme M occurs through two pathways that differ in the steps leading to the production of l-sulfopyruvate, the product of the third step of the canonical pathway catalyzed by ComC [2]. In the originally characterized pathway found in the orders Methanococcales, Methanobacteriales, and Methanopyrales, four enzymes have been characterized thus far [2, 6]. These are phosphosulfolactate synthase (ComA; EC 4.4.1.19), 2-phosphosulfolactate phosphatase (ComB; EC 3.1.3.71) [7], sulfolactate dehydrogenase (ComC; EC 1.1.1.337 (formerly EC 1.1.1.272)) [8], and sulfopyruvate decarboxylase (ComDE; EC 4.1.1.79) [9]. The substrates for ComA are phosphoenolpyruvate and sulfite, and the enzyme produces (*R*)-phosphosulfolactate, which is then dephosphorylated by ComB to produce (*R*)-sulfolactate. ComC oxidizes (*R*)-sulfolactate to sulfopyruvate, which is then decarboxylated by ComDE to produce sulfoacetaldehyde. ComC is related by sequence to lactate/malate dehydrogenases, *N*-methyl-l-amino acid dehydrogenases, 2,3-diketo-l-gulonate reductases, ureidoglycolate dehydrogenases, and an uncharacterized clade of thermophilic archaeal proteins [8, 10–12]. ComC is also likely to participate in the biosynthesis of methanopterin through production of (*S*)-hydroxyglutaric acid, which is a component of methanopterin [11].

When the *Methanocaldococcus jannaschii* genome sequence became available, two malate dehydrogenase genes (MJ1425 and MJ0490) were annotated, and the two enzymes were expressed and characterized by Graupner et al. [11]. Although both enzymes possessed malate dehydrogenase activity (converting oxaloacetate to (*S*)-malate) [13], MJ1425 was identified as likely to be the biologically relevant ComC due to its kinetic parameters (higher *V*_max_ and *V*_max_/*K*_M_ for oxidation of (*R*)-sulfolactate by NAD^+^ to sulfopyruvate) and was classified as a sulfolactate dehydrogenase (EC 1.1.1.337) [10, 11]. More recently, the *M. jannaschii* ComC (MJ1425) has been shown to also catalyze the NAD-dependent oxidation of 2-hydroxy-4-mercaptobutyric acid to 4-mercapto-2-oxobutyric acid, a precursor of the natural product 3-mercaptopropionic acid [14]. 3-mercaptopropionic acid is proposed to act as an alternative coenzyme M. Using a structure-based amino acid alignment, Irimia et al. [10] identified three archaeal homologs to the *M. jannaschii* ComC (MJ1425) that should act as true sulfolactate dehydrogenases. One, assigned as a malate dehydrogenase from *Methanothermus fervidus*, has also been characterized (Mfer0389; MdhI or MdhIII or MDH; [8, 11, 15]). The other two are MTH1205 from *Methanothermobacter thermautotrophicus* and MK0392 from *Methanopyrus kandleri*. Additionally, MdhI from *Methanothermobacter marburgensis* (former species name of *Methanobacterium thermoautotrophicum* strain Marburg; MTBMAc15830) has been characterized and corresponds to MTH1205 [16]. In the alternative pathway, which is predicted to be present in the orders Methanosarcinales and Methanomicrobiales, the steps that lead to sulfopyruvate production are carried out by only two enzymes, the first being cysteate synthase that converts l-phosphoserine to l-cysteate (related to threonine synthase) [2, 6, 17]. An aspartate amino acid transferase then carries out the conversion of cysteate to sulfopyruvate [2], whereby the two coenzyme M pathways converge, with both utilizing ComDE. The final steps in the biosynthesis of coenzyme M, proposed to involve reductive thiolation, have not yet to our knowledge been positively confirmed in any species [1, 7, 18].

Structural analysis of the ComC from the hyperthermophilic methanogen *Methanocaldococcus jannaschii* (MJ1425) has revealed that the NADH binding pocket is not a typical Rossmann-fold type as found in the malate/lactate dehydrogenase family and represents a new class of NADH-based dehydrogenase [10]. In addition, analyses of potential substrates suggest that electrostatic interactions are important for substrate recognition [10]. In the structure which forms a tight dimer and with both monomers contributing to coenzyme binding, the coenzyme is in an extended conformation with the nicotinamide ring placed to enable transfer of the pro-(*S*) hydrogen [10]. Only (*S*)-isomers of substrates are indicated to be converted in the reduction reaction [10].

In this paper, we describe the purification and characterization of ComC from *Methanobrevibacter millerae* SM9, a representative of the dominant species of rumen methanogens [19–21]. Methane emissions from ruminants are responsible for approximately a quarter of total man-made methane emissions, or 12–17% of total global methane emissions, [22] and are increasingly being recognized for their contribution to climate change. As coenzyme M is essential for methanogenesis, its biosynthesis potentially represents a specific methanogen target for inhibitor development to control methane emissions from ruminants. Sulfopyruvate reduction by ComC is NADH-dependent and therefore readily amenable to development as an absorbance-based screening assay for rapidly screening compound libraries.

## 2. Materials and Methods

### 2.1. Materials

Materials including sulfopyruvate, oxaloacetate, *α*-ketoglutarate, NADPH, NADH, 3-(*N*-morpholino)propanesulfonic acid (Mops), 1,3-bis(tris(hydroxymethyl)methylamino)propane or Bis-Tris propane (BTP), tris(2-carboxyethyl)phosphine (TCEP), and dithiothreitol (DTT) were purchased from Sigma-Aldrich (USA). Other common chemicals were obtained from ThermoFisher Scientific (NZ).

### 2.2. Bacteria, Plasmids, and Strains

The ComC gene of *Methanobrevibacter millerae* SM9 [21] was amplified using forward primer 5′-CACCATGAAGATAATGAAGGATAACGAAA and reverse primer 5′-TCATTAATCTTCAAGATAAGAATCTATATC with the reverse primer containing two stop codons. The PCR reaction utilized high-fidelity Hercules II DNA polymerase (1.0 *μ*L; Stratagene, USA) in a 50 *μ*L reaction with 0.2 *μ*M of each primer, 0.3 *μ*M dNTP, approximately 20 ng *M. millerae* SM9 DNA, and 1 × buffer. The PCR cycling parameters had an initial denaturation of 95°C for 2 min, followed by 35 cycles of 94°C for 30 s, 56°C for 30 s, and 68°C for 40 s. The PCR product was purified using agarose DNA electrophoresis and a Wizard SV Gel and PCR kit (Promega, USA). It was then inserted into pET151D using TOPO cloning in chemically competent *Escherichia coli* strain TOP 10F according to the manufacturer's instructions (Invitrogen, USA). Colonies were screened by colony PCR using pET151D T7 forward primer and the ComC reverse primer using 2.5 U *Taq* polymerase (Roche, NZ), and then the recombinant plasmids were isolated using alkaline lysis and purification with a Wizard SV Gel and PCR Clean-up kit (Promega, USA). The plasmid used for expression was sequenced to verify that the gene was in frame and that the sequence was identical to the reference sequence and then transformed into *E. coli* Rosetta 2 cells (Novagen, USA).

### 2.3. Protein Expression and Purification

We followed the methods of Schofield et al. [23] to express ComC in *E. coli* and purify the protein using nickel affinity chromatography, except for the following conditions. The lysis buffer was slightly different (50 mM Tris pH 7.5 containing 1 mM DTT, 300 mM NaCl, 10 mM imidazole, 1% (*v*/*v*) Triton X-100, 20% (*v*/*v*) glycerol, 2 mM CaCl_2_, and 2 mM MgCl_2_), and lysis was performed on ice. Cell debris was removed by centrifugation (17,400*g*, 20 min, 4°C), and the supernatant was filtered (0.8 and 0.22 *μ*m). Buffer was exchanged to 20 mM Mops pH 7.0 containing 2 mM TCEP. Glycerol (10% *v*/*v*) was added to the purified protein; it was snap frozen in liquid nitrogen and stored at −85°C until further use.

### 2.4. Assays of ComC Activity

Spectrophotometric measurements and calculation of initial velocity were performed using a Cary 100 UV-vis spectrophotometer (Agilent Technologies, USA) with a thermostatted cuvette holder, using 1 cm path length quartz cuvettes. The consumption of NADH (366 nm, *ε* 3070 M^−1^ cm^−1^) during the reduction of sulfopyruvate by ComC was monitored. Activity was measured at 37°C. *M. millerae* SM9 was isolated from the rumen of a sheep and has an optimal growth temperature of 38°C [21]. One unit of activity (U) is defined as the conversion of one *μ*mol of NADH to NAD^+^ per minute under standard assay conditions.

Standard assay conditions are 0.08–0.25 *μ*M ComC, 300 *μ*M NADH, 500 *μ*M sulfopyruvate, 400 mM KCl, and 50 mM BTP pH 6.5. The standard assay involved incubation of the above solution without sulfopyruvate at 37°C for 4 min and then initiation of the reaction by the addition of substrate (sulfopyruvate). Kinetics for NADH required variable concentration of the coenzyme (10–500 *μ*M) and 500 *μ*M of sulfopyruvate substrate. Sulfopyruvate kinetics with variable concentration (50–600 *μ*M) were carried out using 400 *μ*M NADH. A concentration of 0.08–0.25 *μ*M ComC in the assay was chosen for accuracy and so as to obtain absorbance changes of about 0.1 to 0.2 per minute. The total volume of all assays was 200 *μ*L. Assays were carried out in triplicate. Kinetic parameters were determined by fitting the data to the Michaelis-Menten equation using GraFit [24].

### 2.5. Molecular Mass Determination

We followed the methods of Schofield et al. [23] to determine the native molecular mass of ComC using gel filtration chromatography. However, the filtered sample of ComC (400 *μ*l) was at a concentration of 1 mg·ml^−1^, and the elution buffer was 50 mM Mops pH 7.0 containing 2 mM TCEP and 0.5 M KCl.

### 2.6. General Methods

We followed the general methods of Schofield et al. [23] for electrophoresis and the determination of protein concentration and pH values of buffers. However, spectrophotometric measurements were performed using a Cary 100 UV-vis spectrophotometer (Agilent Technologies, USA).

## 3. Results and Discussion

In this study, the *M. millerae* SM9 ComC was expressed in *E. coli*, purified using nickel affinity chromatography, and characterized. The apparent molecular mass of ComC was 75 kDa, determined by gel filtration chromatography. As the predicted molecular mass of His-tagged ComC is 40104 Da (376 amino acids), this indicates that ComC is dimeric in solution. Both the *M. jannaschii* ComC (MJ1425) [11] and the *M. fervidus* enzyme (Mfer0389) [15] were also shown to be dimeric.

**Figure 1:**
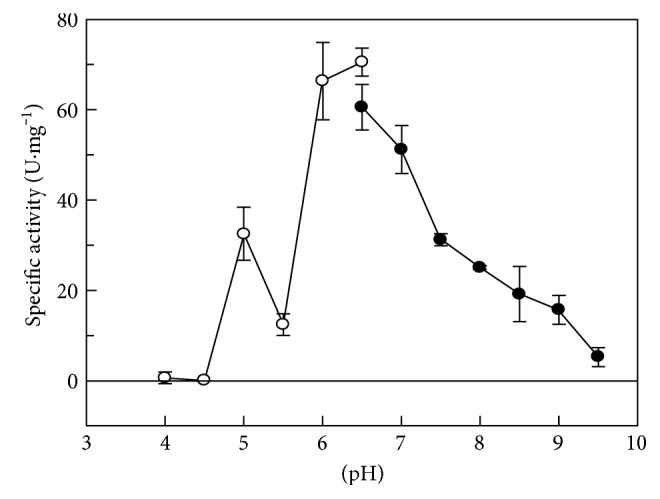
Effect of pH on the specific activity of ComC in the reduction reaction. Two buffers were used; ○ = 50 mM citrate buffer and ● = 50 mM BTP. Other assay conditions were standard; 37°C and 400 mM KCl. Assays were carried out in at least triplicate. One unit of activity (U) is defined as the conversion of one *μ*mol of NADH to NAD^+^ per minute under standard assay conditions.

The effect of pH on the activity of sulfopyruvate reduction by ComC, in the presence of 400 mM KCl, was investigated. Two different buffers (pH 4.0–6.5 citrate buffer and pH 6.5–9.5 BTP buffer) were used. Optimal activity was observed at pH 6.5 (Figure 1). For comparison, the pH used for characterizing the *M. jannaschii* ComC in the reduction reaction was 8.0 [11], the *M. fervidus* enzyme was assayed at pH 7.4 or 8.0 [8, 11, 15], the *M. marburgensis* enzyme (MTBMAc15830) was assayed at pH 7.6 [16] and the *Chromobacter salexigens* ComC was characterized at pH 9.0 [18]. No pH optimization was reported in any of these studies.

**Figure 2:**
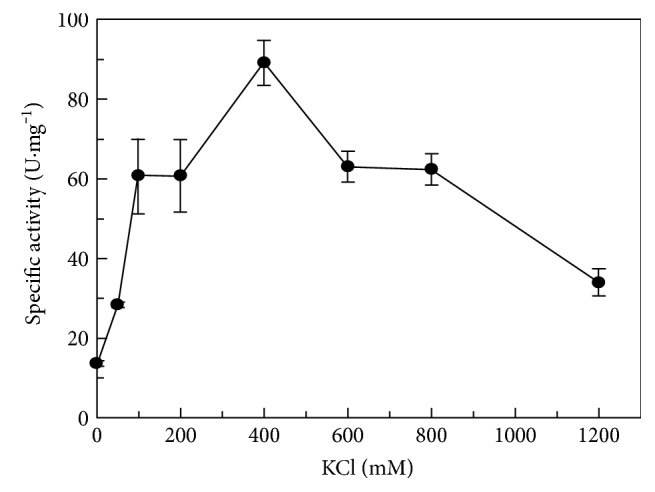
Effect of KCl concentration on the specific activity of ComC in the reduction reaction. Other assay conditions were standard; 37°C and pH 6.5. Assays were carried out in triplicate. One unit of activity (U) is defined as the conversion of one *μ*mol of NADH to NAD^+^ per minute under standard assay conditions.

The activity of sulfopyruvate reduction by ComC at pH 6.5 was affected by the KCl concentration with the enzyme showing low activity in the absence of added KCl. The specific activity increased approximately 7-fold with the increasing KCl concentration in the assay and reached a maximum at 400 mM KCl, after which a progressive decrease in activity was observed (Figure 2). No data on the effect of ionic strength or KCl on ComC has yet been reported to our knowledge. However, the *M. jannaschii* ComC and the *M. fervidus* enzyme were characterized in 100 mM potassium phosphate [11, 15]. High K^+^ and phosphate concentrations, corresponding to the intracellular medium of *M. fervidus* cells, enhanced the thermostability of the *M. fervidus* enzyme [15]. The intracellular concentration of potassium in *M. millerae* SM9 is unknown, but it is not likely to significantly exceed the ionic strength typical for rumen fluid, which is similar to that of blood (0.15 M) [25].

Sulfopyruvate, oxaloacetate, and *α*-ketoglutarate were tested as ComC substrates using the reduction reaction in the presence of NADH. Sulfopyruvate was the preferred substrate, so was used in the standard ComC assay. Oxaloacetate showed 31% of the specific activity seen for sulfopyruvate, and *α*-ketoglutarate showed approximately 0.2%. A similar substrate preference was seen for the *M. jannaschii* ComC which showed a 3-fold lower *K*_M_ for sulfopyruvate compared to oxaloacetate (40 *μ*M and 130 *μ*M, resp.) and a 30-fold higher *V*_max_/*K*_M_ [11]. While for *α*-ketoglutarate, the *M. jannaschii* ComC had a 50-fold lower *K*_M_ (40 *μ*M and 1900 *μ*M, resp.) and a 400-fold higher *V*_max_/*K*_M_ [11]. Furthermore, the *M. fervidus* enzyme had a slightly lower *K*_M_ for sulfopyruvate compared to oxaloacetate and 7-fold higher *V*_max_/*K*_M_ [11].

*M. millerae* SM9 ComC showed less than 1% specific activity in the reduction reaction with NADPH compared to NADH. This bias for NADH was also found for the *M. marburgensis* enzyme which had negligible specific activity using NADPH compared to NADH in the presence of oxaloacetate (<0.4%) [16]. NADH was also preferred by the *M. jannaschii* ComC which showed a 5-fold lower *K*_M_ for sulfopyruvate in the presence of NADH compared to NADPH (40 *μ*M and 210 *μ*M, resp.) and a 60-fold higher *V*_max_/*K*_M_ [11]. Additionally, the *M. fervidus* enzyme had a 3-fold lower *K*_M_ for sulfopyruvate in the presence of NADH compared to NADPH [11] and an almost 3-fold lower *K*_M_ for NADH compared to NADPH in the presence of oxaloacetate [15].

**Figure 3:**
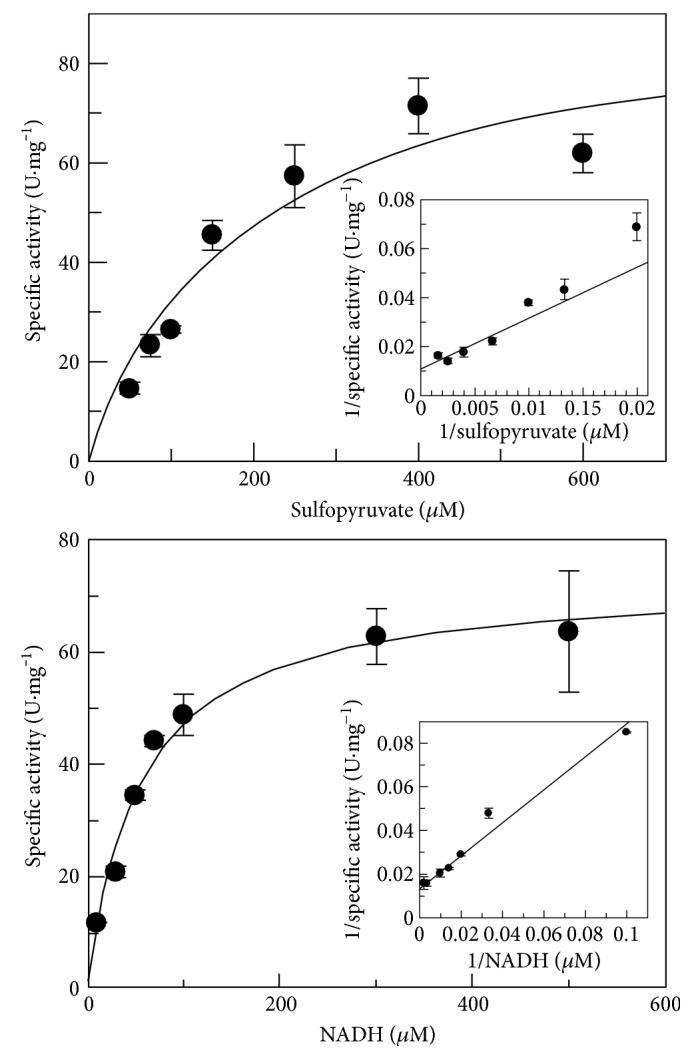
Michaelis-Menten plots for ComC. Michaelis-Menten plots were used to calculate kinetic parameters. The respective Lineweaver-Burk plots are inset. Standard assay conditions in the reduction reaction were used; 400 mM KCl, pH 6.5, and 37°C. Assays were carried out in triplicate. One unit of activity (U) is defined as the conversion of one *μ*mol of NADH to NAD^+^ per minute under standard assay conditions.

The kinetic parameters of the *M. millerae* SM9 ComC (Table 1) were obtained by plots of substrate concentration versus specific activity and their respective double-reciprocal plots (Figure 3). The rates followed typical Michaelis-Menten kinetics for both sulfopyruvate (*K*_M_ 196 *μ*M) and NADH (*K*_M_ 55.1 *μ*M) with the sulfopyruvate value being 5-fold that found for *M. jannaschii* ComC (40 *μ*M) [11]. The *M. millerae* SM9 ComC *K*_M_ for sulfopyruvate is also higher than that for the *M. fervidus* enzyme (70 *μ*M) [11]. The *V*_max_ of the *M. millerae* SM9 ComC for sulfopyruvate was 93.9 U·mg^−1^, and for NADH, it was 73.1 U·mg^−1^. The *V*_max_ value is lower than that for sulfopyruvate of the *M. jannaschii* ComC (370 U·mg^−1^) [11] and similar to that for sulfopyruvate of the *M. fervidus* enzyme (120 U·mg^−1^) [11]. The *M. jannaschii* ComC and the *M. fervidus* enzymes show substrate inhibition at very low sulfopyruvate concentrations (100 *μ*M, i.e., 2.5-fold or 1.4-fold the sulfopyruvate *K*_M_ values of these enzymes, resp.) [11]. The results in Figure 3 may indicate a similar effect for the *M. millerae* SM9 ComC, with possible substrate inhibition at 600 *μ*M, that is, 3-fold the sulfopyruvate *K*_M_ value.

*Methanobrevibacter* is indicated to be the dominant genus of methanogens in the rumen of sheep and cows [19, 20]. Coenzyme M is required for methanogenesis to occur, and therefore targeting enzymes for coenzyme M synthesis represents a valid approach for finding novel small molecule compounds for controlling ruminant methane emissions [20]. Analysis of the genome of coenzyme M-requiring *Methanobrevibacter ruminantium* strain DSM 1093 has revealed, interestingly, that this rumen methanogen does not contain an intact coenzyme M biosynthesis pathway [26]. It contains genes for ComB and ComC but no recognizable genes for ComA or ComDE [26]. Thus, *M. ruminantium* is likely to use coenzyme M derived from other methanogens within the rumen. Lovley et al. [27] have described the isolation of coenzyme-requiring rumen methanogens and those that do not, with the methanogens that can synthesize coenzyme M apparently having faster growth rates.

## 4. Conclusions

We have reported here the cloning, expression, and biochemical characterization of ComC from a representative rumen methanogen. The activity of *M. millerae* SM9 ComC was assayed in the reverse reaction of coenzyme M biosynthesis using sulfopyruvate. The only other ComC to have been extensively characterized is from the thermophilic methanogen *M. jannaschii* [8, 11]. Due to its role in two separate cofactor biosynthesis pathways and its dependence on NADH, the *M. millerae* SM9 ComC represents a potential means of screening compounds for their ability to inhibit methanogens and in so doing, help mitigate methane emissions from ruminants.

## Figures and Tables

**Table 1:** Kinetic parameters for ComC, for sulfopyruvate, and for NADH. Standard assay conditions in the reduction reaction were used; 400 mM KCl, pH 6.5, and 37°C. Assays were carried out in triplicate. One unit of activity (U) is defined as the conversion of one *μ*mol of NADH to NAD^+^ per minute under standard assay conditions.

	*K*_M_ (*μ*M)	*V*_max_ (U·mg^−1^)	*k*_cat_ (s^−1^)
Sulfopyruvate	196 ± 71	93.9 ± 14.5	62.8 ± 9.7
NADH	55.1 ± 7.6	73.1 ± 3.2	48.9 ± 2.2
